# Expert‐validated criteria for evaluating 2D anatomical images: Results of a Delphi consensus study

**DOI:** 10.1002/ase.70301

**Published:** 2026-08-03

**Authors:** Ayşe Soylu, Yavuz Selim Kıyak, Özlem Coşkun, Tuncay Veysel Peker

**Affiliations:** ^1^ Department of Anatomy, Faculty of Medicine Gazi University Ankara Turkey; ^2^ Department of Medical Education and Informatics, Faculty of Medicine Gazi University Ankara Turkey

**Keywords:** 2D visual, anatomy education, Delphi method, expert consensus, visual material

## Abstract

Currently, there are no consensus standards for the selection of two‐dimensional (2D) anatomical images that form the basis of anatomy education. The absence of standardized evaluation criteria may contribute to variability in image quality and inconsistencies in the educational use of anatomical visuals, potentially affecting students' development of an accurate understanding of anatomy. Addressing this gap is important for supporting the reliable and educationally effective use of 2D anatomical images in undergraduate medical education. An initial pool of 70 candidate criteria was generated through literature review and expert input and organized into seven domains. A modified two‐round Delphi process was conducted. In Round 1 (*n* = 20), experts rated each criterion on a five‐point scale. Domain‐specific cut‐off values were applied, and the item‐level content validity index (I‐CVI) was calculated. In Round 2 (*n* = 15), experts reviewed domain appropriateness and item wording. Thirty‐four criteria met predefined thresholds and were retained. The scale‐level content validity index was high (S‐CVI/Ave = 0.94). In Round 2, mean agreement on domain appropriateness was 97.8%, and 90.2% of items were retained without modification. Modified kappa analysis demonstrated excellent agreement beyond chance (mean k* = 0.93). No new criteria were proposed. This study developed a 34‐item, expert‐validated checklist for evaluating static 2D anatomical images. The high consensus levels support its potential utility in improving the quality, consistency, and educational relevance of visual materials used in anatomy education.

## INTRODUCTION

Static two‐dimensional (2D) anatomical images, defined as non‐animated, planar visual representations of anatomical structures, are indispensable tools in medical education because they can convey complex spatial information. The complex spatial organization of anatomical structures presents a substantial challenge for learners, making text‐based instruction alone insufficient in anatomy education. Visual materials, therefore, represent an essential component of anatomy teaching because they support spatial understanding, clarify structural relationships, and facilitate the application of anatomical knowledge in clinical contexts.^1^, ^2^, ^3^ Incorporating diverse visual materials, including clinical and radiological images, further supports integrated anatomy learning and helps learners connect anatomical knowledge with clinical practice, thereby enhancing clinical reasoning.^4^, ^5^ The quality of these visuals also plays an important role in learning outcomes. In accordance with the principles of Multimedia Learning Theory, students demonstrate improved recall and comprehension when textual information is combined with high‐quality visual materials rather than presented as text alone.^6^ Similarly, Cognitive Load Theory suggests that anatomically accurate, well‐organized, and visually clear images can reduce extraneous cognitive load and support meaningful learning processes by facilitating efficient processing of complex spatial information.^7^ Accordingly, well‐designed visuals facilitate more effective interpretation of anatomical relationships, enhance students' spatial comprehension and learning effectiveness, and may positively influence assessment performance in anatomy education.^2^, ^6^, ^7^ In contrast, poor‐quality or inaccurately designed visuals may lead to misinterpretation and increased cognitive load.^2^, ^8^, ^9^

A growing body of literature demonstrates that visual learning improves academic performance, spatial perception, and assessment performance in anatomy education.^2^, ^10^ Accordingly, a wide range of visual resources (e.g., atlas illustrations, cadaveric photographs, and radiological images) are commonly used in anatomy curricula. The integration of clinical and radiological visuals has been shown to increase student engagement and support clinical reasoning.^3^, ^11^ However, despite the demonstrated educational value of visuals, systematic and applicable guidelines for selecting, evaluating, and presenting anatomy images remain limited.^3^, ^12^

Anatomy educators frequently face challenges in identifying high‐quality visuals that are both anatomically accurate and pedagogically appropriate.^13^, ^14^ Variability in image quality, resolution, labeling, and anatomical accuracy directly affects learning and assessment outcomes.^2^, ^3^, ^10^ In the absence of standardized criteria, educators often rely on personal experience and subjective judgment when selecting visual materials. Although prior research has compared various teaching methods and visual formats, it has not yet produced a comprehensive framework that defines the essential components of a “high‐quality anatomical image”.^13^, ^15^

Recently, there has been an increase in studies evaluating the educational quality and anatomical accuracy of AI‐based visualizations. For example, Eldesoqui et al. compared the output of four different AI text‐image generators and found frequent errors in skeletal structures. Alon et al. reported widespread representational bias and clinical accuracy issues in a systematic review encompassing 31 empirical studies.^16^, ^17^ These findings highlight both the potential and risks of AI‐generated visualizations and reinforce the need for standardized, expert‐validated assessment frameworks. However, current research largely relies on self‐developed checklists or ad hoc tools that are not systematically developed, supported by expert opinion, or formally validated. This limits the reliability and comparability of the findings, particularly given the potential for subtle anatomical errors in AI‐generated visualizations. The absence of rigorously developed evaluation criteria further underscores the need for standardized, expert‐validated frameworks applicable to both traditional and novel visual sources. This gap is well documented in the literature.^12^, ^17^, ^18^, ^19^, ^20^

In this context, the Delphi method offers a structured approach to consensus that systematically collects expert opinions through iterative feedback rounds. Its anonymous and multi‐round structure reduces individual bias and makes it particularly suitable for developing criteria in areas where empirical evidence is limited.^35,21^ Therefore, the Delphi method is widely used to gather reliability and validity evidence for consensus‐based frameworks in educational research.

This study aimed to develop an expert‐validated consensus‐based checklist for evaluating static 2D anatomical images. We systematically combined expert opinions using the Delphi method to develop a standardized method for evaluating static 2D images. The goal of this checklist was to improve content quality, anatomical accuracy, and pedagogical consistency in anatomy education.

## METHOD

### Research design

The Delphi method is particularly well‐suited for systematically eliciting and synthesizing expert judgments in domains where standardized, evidence‐based guidance is scarce. Because this study addresses a complex educational field lacking established evaluation criteria and requiring the iterative integration of multidisciplinary perspectives, the Delphi approach was considered appropriate for achieving expert consensus. Accordingly, a modified two‐round Delphi design was implemented.^22^ The study adopted a structured methodological framework grounded in iterative expert review conducted through online surveys. As the primary objective was to establish content validity using the Delphi method, comprehensive construct validity testing was beyond the scope of this initial Delphi‐based content validation study.

Ethics approval for the planning and data collection phases was obtained from the Gazi University Ethics Commission (Research Ethics Committee Code No: 2025‐1651). Participation was voluntary, and informed consent was obtained from all participants whose identities were kept confidential.

### Participants

A purposive sampling strategy was used to establish the expert panel. The selection process was conducted collaboratively by a multidisciplinary research team comprising members from the Departments of Anatomy and Medical Education, ensuring perspectives from both anatomy education and educational methodology. The Department of Anatomy contributed expertise in anatomical visualization and undergraduate anatomy teaching. The Department of Medical Education and Informatics provided expertise in instructional design, digital learning, and AI‐supported educational technologies. This multidisciplinary approach facilitated the identification of experts with complementary educational and disciplinary expertise relevant to evaluating anatomical visuals used in undergraduate medical education.

To enhance institutional diversity, experts were recruited from multiple universities and health sciences faculties. Participants represented a range of institutional settings and academic ranks, providing diverse perspectives on anatomy education, anatomical visualization, and educational resource development. The expert panel comprised academics and professionals actively involved in undergraduate anatomy education. Inclusion criteria were: (i) academic training and professional experience in anatomy or anatomy education, and (ii) active involvement in undergraduate anatomy teaching at a medical or health sciences institution. Experts were purposively selected based on their demonstrated experience in anatomy teaching, anatomical visualization, curriculum development, educational material preparation, and/or anatomy‐related research. The panel represented a range of academic ranks and educational backgrounds, ensuring broad expertise relevant to the evaluation of anatomical visuals used in undergraduate medical education. Previous Delphi studies have reported that expert panels of 10–15 participants are sufficient for achieving reliable consensus.^21^, ^23^ Experts were contacted directly and informed of the study's purpose, scope, and procedures.

### Criteria pool and domain classification

The multidisciplinary research team generated an initial pool of 70 candidate criteria based on a review of the anatomy education literature and the research team's expertise in anatomy teaching, assessment, and instructional design. The literature review was conducted by the research team using databases such as PubMed, Google Scholar, and Scopus, utilizing search terms including “anatomy education,” “visual materials,” “image quality,” “2D visuals,” and “multimedia learning.” The initial findings were then synthesized through structured discussions within the multidisciplinary research team to formulate the criteria, consistent with established approaches in Delphi‐based research.^24^, ^25^ Criteria were formulated as clear, single‐concept statements suitable for checklist‐based evaluation. During checklist development, conceptually related criteria were intentionally retained as separate items when they were considered to represent distinct perceptual, technical, or pedagogical dimensions of anatomical image evaluation. For example, image resolution, linework/clarity, and overall image quality were treated as separate constructs because each addresses a different aspect of image interpretation and educational usability.

The candidate criteria were grouped into seven predefined domains:

- Technical and Visual Quality

Refers to the clarity, resolution, and overall visual fidelity of images, which are essential for accurate perception and effective learning in anatomy education.^2^

B Layout, Composition, and Organization

Encompasses the spatial arrangement and visual organization of elements, which influence attention guidance and comprehension in visual learning environments.^6^

C Labels and Typography

Addresses the accuracy, readability, and consistency of labels and text, which are critical for integrating visual and verbal information and reducing cognitive load.

D Colors, Light, and Shading

Relates to the use of color, contrast, and illumination to enhance discrimination of structures and highlight relevant information.^24^

E Instructional Design

Refers to the extent to which an image is educationally meaningful, appropriately focused, and capable of supporting the intended learning task. This domain includes considerations such as highlighting relevant anatomical information, minimizing unnecessary visual complexity, promoting learner engagement, and facilitating understanding of anatomical concepts in educational contexts.^6^

F Anatomical Accuracy

Refers to the correctness of structure, proportion, spatial relationships, and terminology, which is fundamental to preventing misconceptions in anatomy learning.^26^

G Documentation and Ethics

Includes proper attribution, source transparency, and adherence to copyright and ethical standards in educational materials.

This domain structure was established before the Delphi process and used consistently throughout both rounds.

### Delphi process and data collection tool

Data were collected using anonymous online questionnaires administered via Google Forms. All responses were kept anonymous from other panelists and the research team during analysis.

#### Round 1

In the first round, experts evaluated each of the 70 candidate criteria using a 5‐level ordinal scale (1 = must be removed; 2 = should be removed; 3 = undecided; 4 = should be included; 5 = must be included). Experts could also suggest additional criteria or provide comments using open‐ended response fields. After Round 1, criteria were evaluated using predefined, domain‐specific cut‐off points determined by the internal team via consensus. The cut‐off values are presented in Table 2.

In a systematic review of Delphi studies,^27^ Diamond et al. reported that percentage agreement was the most commonly used definition of consensus and that 75% (3.75 out of 5) was the median threshold among studies using this approach. Stricter thresholds (e.g., ≥4.5) were applied to domains considered foundational for safe and accurate anatomy learning (e.g., anatomical accuracy and technical quality) to enforce a high level of consensus. In comparison, slightly lower thresholds (e.g., 4.0 or 4.25) were used for domains where expert judgment may be more context‐dependent or inherently involves context‐dependent visual interpretation (e.g., colors, light, and shading). These domain‐specific cut‐off points were established a priori by the research team to maintain methodological rigor while accommodating the nuanced nature of visual evaluation. Criteria with mean scores below the relevant cut‐off point were eliminated. Criteria with mean scores below the relevant cut‐off point were eliminated. The remaining criteria were subsequently evaluated in Round 2. For each item, a mean importance score was calculated from expert ratings on a 5‐point Likert scale. Domain‐specific cut‐off points were then applied, and items meeting or exceeding the predefined threshold were retained for subsequent Delphi rounds.

#### Round 2

In the second round, experts evaluated only the reduced list of 34 criteria. To minimize conformity bias, statistical summaries from Round 1 were not shared with participants during Round 2. For each criterion, participants were asked to indicate: (i) whether the criterion was appropriate for the domain to which it was assigned (yes/no), and (ii) their recommendation regarding the item wording (“can be retained as is,” “should be retained with revised wording,” or “should be removed entirely”).

For each criterion, participants selected one of three response options regarding item retention: (1) retain as written, (2) retain with revised wording, or (3) remove entirely. A criterion was considered to have achieved consensus if at least 90% of participants selected one of the two retention options (retain as written or retain with revised wording). Criteria failing to meet this threshold would have been excluded from the final checklist. All criteria met this predefined threshold in Round 2; therefore, no additional Delphi rounds were required. Minor wording revisions were incorporated based on expert feedback.

### Statistical analysis

Analyses were performed using Microsoft Excel. In Round 1, response frequencies, percentages, means (standard deviations), medians, and interquartile ranges (IQRs) were calculated for each criterion. Although the response scale was ordinal, mean scores were used pragmatically to support ranking and application of domain‐based cut‐off points, consistent with standard practice in Delphi studies; medians and IQRs were also reported to describe response distributions.

Content validity was assessed using the item‐level content validity index (I‐CVI), calculated as the proportion of experts who rated an item 4 (“should be included”) or 5 (“must be included”). For the final list, the scale‐level content validity index (S‐CVI/Ave) was calculated as the mean of the I‐CVI values across all retained items. To account for agreement occurring by chance, modified kappa statistics (k*) were calculated for retained items following the method proposed by Polit, Beck, and Owen.^28^ The probability of chance agreement (Pc) was first estimated and subsequently used to calculate k* values according to the formula: k* = (I‐CVI − Pc)/(1 − Pc). Modified kappa values were interpreted as follows: >0.74 = excellent agreement, 0.60–0.74 = good agreement, and 0.40–0.59 = fair agreement.

In Round 2, results were summarized using percentages for domain appropriateness and wording recommendations. Open‐ended comments were reviewed qualitatively to inform minor wording revisions. Because the primary aim of Delphi studies is consensus development, no inferential statistical tests were applied, and results were reported descriptively.

## RESULTS

### Participant characteristics

Twenty experts participated in Round 1, including 13 professors, 3 assistant professors, and 4 lecturers who were actively involved in undergraduate anatomy education. The expert panel represented expertise in anatomy education and medical education. Of the 20 experts who completed Round 1, 15 also completed Round 2, corresponding to a retention rate of 75% and an attrition rate of 25% between rounds. Participant characteristics are presented in Table 1. The overall Delphi process, including participant retention and criterion selection across rounds, is summarized in Figure 1.

**TABLE 1:** Number of participants and purpose by Delphi round.

Round	Number of participants (*n*)	Purpose
Round 1	20	Evaluation of candidate criteria (importance/inclusion)
Round 2	15	Review of domain appropriateness and wording of the remaining criteria

**FIGURE 1:**
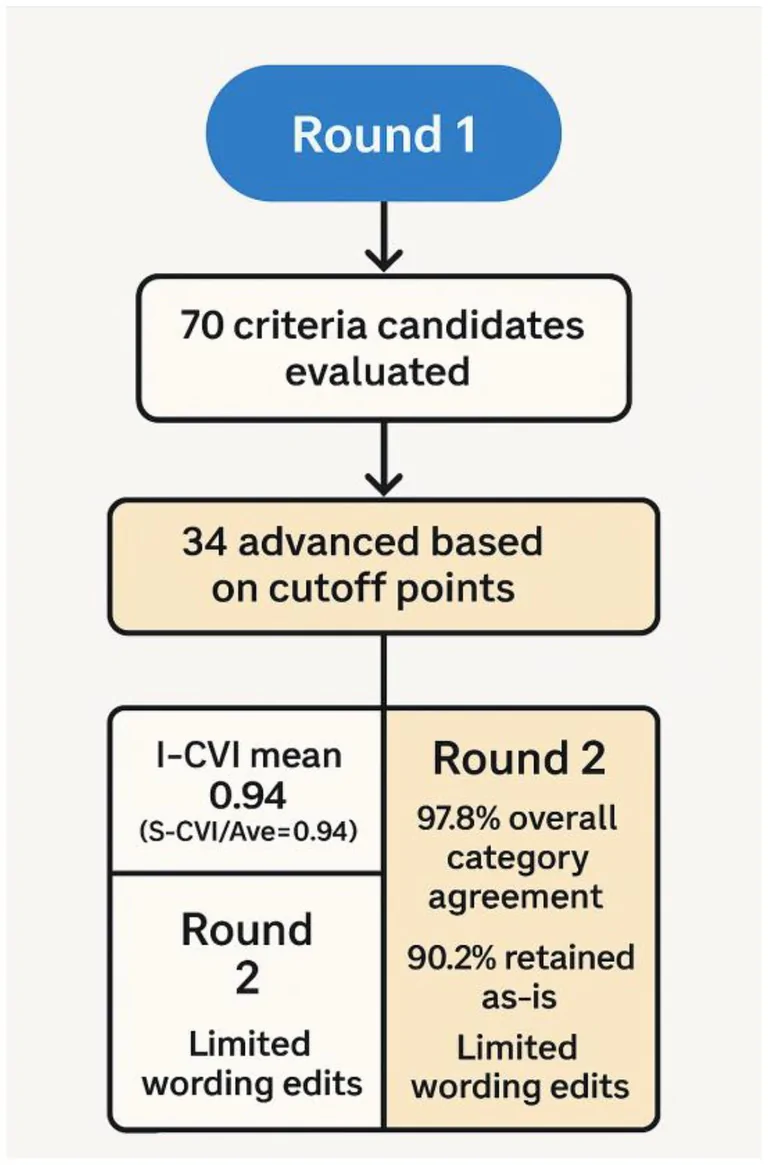
A two‐round Delphi process was used to select and refine the criteria. In Round 1, 70 candidate criteria were evaluated, and 34 criteria advanced based on predefined cutoff points. The mean item‐level content validity index (I‐CVI) was 0.94, with an overall scale‐level CVI (S‐CVI/Ave) of 0.94. In Round 2, overall category agreement reached 97.8%, and 90.2% of the criteria were retained without modification. Only limited wording edits were required.

### Round 1 outcomes

Application of the predefined domain‐specific cut‐off values resulted in the retention of 34 of the initial 70 candidate criteria (48.6%) for further evaluation in Round 2. The criteria receiving the highest mean ratings are primarily related to Technical and Visual Quality and Anatomical Accuracy, particularly image quality and resolution. The highest number of retained criteria belonged to the Anatomical Accuracy domain (11 of 15 items), followed by Technical and Visual Quality (7 of 13 items) and Labels and Typography (5 of 9 items) (Table 2). For the retained criteria, item‐level content validity index (I‐CVI) values ranged from 0.70 to 1.00 (median = 0.95), and 15 criteria achieved perfect agreement (I‐CVI = 1.00).

**TABLE 2:** Domain‐specific retention thresholds, item retention outcomes, and cut‐point justifications.

Domain	Initial items	Retained (*n*/*N*)	Cut‐off point
A. Technical and Visual Quality	10	7/10	4.5
B. Layout, Composition, and Organization	7	3/7	4.5
C. Labels and Typography	10	5/10	4.5
D. Colors, Light, and Shading	9	3/9	4.25
E. Instructional Design	10	2/10	4.5
F. Anatomical Accuracy	20	11/20	4.5
G. Documentation and Ethics	4	3/4	4.0

*Note*: Domain‐specific cut‐off values were established a priori based on the perceived educational importance of each domain and the level of expert consensus considered necessary for item retention. Higher thresholds (≥4.50) were applied to domains regarded as fundamental for the accurate interpretation and educational use of anatomical images, including Anatomical Accuracy, Technical and Visual Quality, Labels and Typography, and Instructional Design. These domains were considered critical because deficiencies in these areas may directly affect anatomical understanding, image interpretation, and educational effectiveness. Lower cut‐off thresholds were used for domains involving greater contextual variability or subjective judgment.

### Round 2 outcomes

The final checklist consisted of 34 criteria distributed across seven domains (Table 3). The 34 retained criteria were re‐evaluated for both domain appropriateness and wording. Mean agreement regarding domain appropriateness was 97.8%. The mean recommendation rate for retaining criteria without modification was 90.2%. Minor wording revisions were suggested for nine criteria, whereas no additional criteria were proposed by panel members. All criteria exceeded the predefined retention threshold and were therefore retained in the final checklist. Consequently, no additional Delphi rounds were required.

**TABLE 3:** Final criteria list (*n* = 34) and Round 1 content validity indicators.

Domain	Item text	Median (IQR)	I‐CVI
A. Technical and Visual Quality	Linework/clarity should be sufficient	5 (1)	1.00
A. Technical and Visual Quality	Resolution should allow 100% magnification without blurring	5 (1)	1.00
A. Technical and Visual Quality	Image size should be easily perceivable	5 (1)	0.95
A. Technical and Visual Quality	Watermarks should not obstruct structures	4.5 (1)	1.00
A. Technical and Visual Quality	Image resolution should be sufficient	5 (0.25)	1.00
A. Technical and Visual Quality	Overall image quality should be sufficient	5 (0)	1.00
A. Technical and Visual Quality	Structures should be drawn at a perceivable size	5 (0.25)	0.95
B. Layout, Composition, and Organization	Distracting background textures should be avoided	5 (1)	1.00
B. Layout, Composition, and Organization	Adequate contrast should exist among structures and labels	5 (1)	1.00
B. Layout, Composition, and Organization	Perspective and angle should be appropriate	4.5 (1)	1.00
C. Labels and Typography	Typography should be consistent	5 (1)	0.90
C. Labels and Typography	Labels should be accurate and complete	5 (1)	1.00
C. Labels and Typography	Labels should clearly indicate structures	5 (0)	1.00
C. Labels and Typography	Typography should be readable	5 (1)	0.95
C. Labels and Typography	Structures should be named correctly	5 (0)	1.00
D. Colors, Light, and Shading	Colors should highlight important information	4 (1)	0.80
D. Colors, Light, and Shading	Lighting and contrast should distinguish structures	4 (1)	0.90
D. Colors, Light, and Shading	Colors should be clear and vivid	5 (1.25)	0.75
E. Instructional Design	Image should clearly present emphasized structures	5 (0)	1.00
E. Instructional Design	Image should contain sufficient detail	5 (1)	0.90
F. Anatomical Accuracy	No perspective errors should exist	5 (1)	0.90
F. Anatomical Accuracy	Structures should be distinct and clear	5 (1)	1.00
F. Anatomical Accuracy	Section planes should be correct	5 (1)	0.95
F. Anatomical Accuracy	Terminology should follow international standards	5 (0)	0.95
F. Anatomical Accuracy	Adjacency relationships should be correct	5 (0)	1.00
F. Anatomical Accuracy	Structures should be proportionate	5 (0.25)	0.90
F. Anatomical Accuracy	Structures should be positioned correctly	5 (1)	1.00
F. Anatomical Accuracy	Image should not omit normal anatomy	5 (1)	0.85
F. Anatomical Accuracy	Image should contain no anatomical errors	5 (0)	0.95
F. Anatomical Accuracy	Directional indicators should be correct	5 (0.25)	0.95
F. Anatomical Accuracy	Symmetry/asymmetry should be considered	5 (1)	0.90
G. Documentation and Ethics	Production source/method should be stated	4 (2)	0.70
G. Documentation and Ethics	References/source materials should be stated	4 (1)	0.80
G. Documentation and Ethics	Copyright/licensing should be clear	4 (1)	0.85

To account for agreement occurring beyond chance, modified kappa (k*) coefficients were calculated for the retained criteria. The mean k* value was 0.93, indicating excellent agreement beyond chance. Thirty‐two criteria demonstrated excellent agreement (k* ≥ 0.74), whereas two demonstrated good agreement (k* = 0.60–0.74).

### Stability of responses across Delphi rounds

Because responses were collected anonymously and individual participants could not be linked across Delphi rounds, intra‐rater reliability analyses could not be performed. Consequently, stability of individual responses between rounds could not be assessed directly. Instead, consistency of expert consensus was evaluated using multiple complementary indicators, including percentage agreement, item‐level content validity indices (I‐CVI), scale‐level content validity indices (S‐CVI/Ave), and modified kappa coefficients (k*).

## DISCUSSION

### Overview of findings

This study developed a structured, expert–consensus–based checklist for evaluating static two‐dimensional (2D) anatomy visuals used in health profession education. Through a two‐round Delphi process, 34 criteria across seven domains were retained from an initial pool of 70 candidate items, reflecting a balance between comprehensiveness and practical usability. The high level of agreement achieved in Round 2 (mean domain appropriateness: 97.8%) and the strong content validity indices (S‐CVI/Ave = 0.94) support the methodological rigor and content relevance of the resulting framework.

### The central role of anatomical accuracy and technical quality

The predominance of criteria related to anatomical accuracy (*n* = 11) and technical and visual quality (*n* = 7) indicates that experts consider anatomical correctness and visual clarity to be the foundational determinants of image quality in anatomy education. Inaccuracies in proportions, spatial relationships, labeling, or structural representation may contribute to persistent misconceptions and introduce construct‐irrelevant variance in assessment contexts, particularly when visuals are incorporated into examinations.^10^ The concentration of retained items within these domains suggests a shared expert perspective that visual attractiveness alone is insufficient; anatomical fidelity and technical clarity are essential for safe, effective, and educationally meaningful learning experiences. Although anatomical accuracy and technical and visual quality emerged as the domains with the highest number of retained criteria, the ordering of domains within the checklist reflects the original conceptual framework established before the Delphi process rather than a ranking of relative importance.

### Visual–verbal integration and learning

The inclusion of multiple criteria addressing labels and typography further emphasizes the importance of visual–verbal integration in anatomy teaching. Clear, consistent, and readable labeling helps learners identify structures accurately and reduces extraneous cognitive load. From a cognitive load theory perspective, poorly organized or inconsistently labeled visuals may divert mental resources away from learning toward deciphering the image itself.^7^, ^12^, ^29^ Similarly, principles from multimedia learning theory suggest that coherent alignment of visual and verbal information facilitates deeper learning and comprehension. The retention of these criteria, therefore, reflects pedagogical considerations rather than purely aesthetic concerns.

### Educational implications for instructional design

Although fewer criteria were retained in the instructional design domain (*n* = 2), it remains pedagogically important. The retained criteria emphasize clarity of instructional intent and sufficient anatomical detail, underscoring that even high‐quality images must be aligned with clearly defined learning objectives. In this context, instructional intent does not refer to explicit learning objectives accompanying an image. Rather, it refers to whether the image appears suitable for the educational purpose for which it is selected (e.g., introductory learning, detailed anatomical study, or clinical application). Although the present study focused primarily on image selection rather than instructional deployment, the proposed checklist may also assist educators in planning how anatomical visuals are incorporated into teaching and assessment activities, particularly in visual‐based examinations, where image characteristics may influence item difficulty and discrimination.^10^ From a practical perspective, the checklist is not intended to require exhaustive evaluation of every image contained in a large anatomy course repository. Instead, it may be most useful as a targeted quality assurance tool for high‐stakes or high‐impact visuals, such as images used in examinations, core teaching sessions, newly developed instructional materials, or resources selected for digital learning environments. Educators may also apply the checklist in a staged manner, first screening images for essential criteria related to anatomical accuracy and technical quality and then evaluating additional domains such as labeling, layout, instructional suitability, and documentation when more detailed review is required.

### Practical implementation of the checklist

To implement this checklist effectively, it should be used as a reflective, improvement‐oriented tool rather than a rigid evaluative framework.^24^, ^30^ It is recommended that educators rate the checklist statements using a simple categorical scale (e.g., “Meets,” “Partially Meets,” or “Does Not Meet”) rather than a strict numerical score, consistent with established rubric‐based assessment approaches.^31^, ^32^ To mitigate subjective bias in criteria such as “Linework/clarity should be sufficient,” future implementations would benefit from developing standardized scoring rubrics and providing rater training.^29^ When evaluating images that yield variable scores across domains, a hierarchical approach is advised, prioritizing essential domains such as anatomical accuracy and technical quality as prerequisites for educational use. In cases where images meet core criteria but fall short in secondary domains (e.g., layout or typography), educators may mitigate these limitations through instructional scaffolding, such as verbal clarification or supplementary annotations, consistent with principles of cognitive load and multimedia learning.^6^, ^7^ The checklist may also serve different purposes depending on the intended context of use. For example, images selected for routine educational activities may require satisfaction of core anatomical and technical criteria, whereas images intended for publication, assessment development, or dissemination as educational resources may warrant evaluation across all domains.

Future research should investigate the feasibility of weighted scoring systems, domain‐specific thresholds, and context‐dependent standards for educational and publication purposes.

### Relevance for digital and AI‐generated anatomical visuals

The relevance of standardized selection criteria is further heightened in contemporary digital learning environments. As anatomy education increasingly relies on digital resources, variability in resolution, scaling, and display conditions becomes more consequential. High‐resolution images that retain clarity under magnification are essential for supporting detailed anatomical inspection, particularly in self‐directed learning contexts. Moreover, the checklist may be especially valuable for evaluating AI‐generated anatomical images,^16^, ^18^, ^19^, ^20^ which can appear visually convincing yet contain subtle anatomical inaccuracies or proportional errors that may not be readily apparent to learners. Beyond post‐generation evaluation, the checklist components can be proactively utilized as a structured framework for prompt engineering. Educators can incorporate these specific criteria (e.g., requiring correct adjacency relationships, accurate section planes, and appropriate lighting) into their prompts to design higher‐quality AI‐generated anatomical content, consistent with emerging approaches in prompt engineering and AI‐assisted content generation.^33^, ^34^ This approach may help mitigate known risks of anatomical inaccuracies in AI‐generated visuals while improving educational relevance.^6^, ^7^ The absence of rigorously developed evaluation frameworks in prior studies assessing AI‐generated anatomy visuals underscores the importance of expert‐validated criteria that can be applied consistently across traditional and emerging image sources.

### Implications for assessment quality

An additional strength of the checklist lies in its applicability to evaluate visual‐based assessment materials. Images used in anatomy examinations play a critical role in directing student attention and shaping task demands. Variations in image quality, labeling, or anatomical representation can inadvertently alter item difficulty and threaten assessment validity.^10^, ^15^ By providing standardized criteria for image selection, the checklist offers a potential mechanism to reduce unintended construct‐irrelevant variance in image‐based assessments.

### Limitations

Several limitations should be considered when interpreting these findings. The expert panel was formed using purposive sampling within a single national context, which may limit generalizability. Although consensus thresholds were achieved for all retained items, the two‐round modified Delphi design may have limited opportunities for additional refinement. The study focused exclusively on static 2D anatomical visuals. Therefore, the applicability of the proposed criteria to interactive, three‐dimensional, or animated resources was not evaluated. Although strong content validity was established, other psychometric properties, including construct validity, inter‐rater reliability, pilot implementation, and relationships with student learning outcomes, were not assessed.

In addition, responses were collected anonymously and were not linked across Delphi rounds. Consequently, the stability of individual expert judgments over time could not be evaluated, and intra‐rater reliability analyses were not feasible. Although anonymity was intentionally employed to promote independent expert judgment and reduce conformity bias, future Delphi studies may benefit from the use of participant identifiers that preserve confidentiality while allowing assessment of response stability across rounds.

Because Delphi‐based consensus processes prioritize expert‐identified dimensions, some degree of conceptual overlap among retained items may persist. Future psychometric studies, including exploratory and confirmatory factor analyses, may help determine whether some criteria can be combined, weighted differently, or organized into a more parsimonious dimensional structure while preserving their educational relevance.

### Future directions

Future studies should evaluate the checklist's validity and reliability across different educational settings and institutions, including pilot implementation and inter‐rater agreement analyses. Additional research should also investigate whether checklist‐guided image selection improves learning outcomes, reduces cognitive load, and enhances the quality of visual‐based assessments.

The rapid emergence of artificial intelligence presents several additional opportunities for future research. Studies should explore whether large language models (LLMs) and AI‐assisted evaluation systems can reliably apply the checklist to large collections of anatomical images and thereby improve the scalability of image quality assessment. Comparative investigations could evaluate traditional anatomical illustrations (e.g., textbook and atlas images) alongside AI‐generated visuals using the checklist as a standardized evaluation framework. Future research may also examine how checklist criteria can be incorporated into prompt engineering strategies to improve the anatomical accuracy, educational suitability, and visual quality of AI‐generated content. Furthermore, studies are needed to determine whether the checklist can be adapted for use with interactive, three‐dimensional, virtual reality, augmented reality, and animated anatomical learning resources. Collectively, these research directions may contribute to the development of evidence‐based standards for both the evaluation and generation of anatomical images in increasingly AI‐supported educational environments.

## CONCLUSION

This two‐round Delphi study developed a standardized 34‐item checklist for evaluating the quality of 2D anatomical images. High agreement among experts and strong content validity indices suggest the checklist has reasonable content validity. The checklist was developed to help educators select anatomically accurate, pedagogically appropriate, and high‐quality visuals. However, additional research is needed to gather further evidence of the tool's reliability and construct validity to ensure its functional utility. Upon further validation, its integration into instructional design and assessment practices may help establish shared quality standards and enhance the overall effectiveness of visual materials used in anatomy education.

## AUTHOR CONTRIBUTIONS

**Yavuz Selim Kıyak:** Methodology; conceptualization; writing – review and editing; resources. **Tuncay Veysel Peker:** Methodology; conceptualization; writing – review and editing; data curation; project administration; writing – original draft; resources. **Özlem Coşkun:** Methodology; conceptualization; writing – review and editing; data curation; project administration; writing – original draft; resources. **Ayşe Soylu:** Conceptualization; methodology; data curation; writing – review and editing; formal analysis; writing – original draft; resources.

## CONFLICT OF INTEREST STATEMENT

There is no conflict of interest.

## ETHICS APPROVAL

This study was approved by the Gazi University Institutional Review Board (Approval No: 2025‐1651).

## Data Availability

The data that support the findings of this study are available from the corresponding author upon reasonable request.
